# A case-control study to evaluate the impact of the breast screening programme on mortality in England

**DOI:** 10.1038/s41416-020-01163-2

**Published:** 2020-11-23

**Authors:** Roberta Maroni, Nathalie J. Massat, Dharmishta Parmar, Amanda Dibden, Jack Cuzick, Peter D. Sasieni, Stephen W. Duffy

**Affiliations:** 1 Centre for Cancer Prevention, Wolfson Institute of Preventive Medicine, Queen Mary University of London, Charterhouse Square, London, EC1M 6BQ UK; 2 Faculty of Life Sciences and Medicine, Cancer Prevention Group, School of Cancer and Pharmaceutical Sciences, King’s College London, Guy’s Campus, Great Maze Pond, London, SE1 9RT UK

**Keywords:** Cancer screening, Breast cancer

## Abstract

**Background:**

Over the past 30 years since the implementation of the National Health Service Breast Screening Programme, improvements in diagnostic techniques and treatments have led to the need for an up-to-date evaluation of its benefit on risk of death from breast cancer. An initial pilot case-control study in London indicated that attending mammography screening led to a mortality reduction of 39%.

**Methods:**

Based on the same study protocol, an England-wide study was set up. Women aged 47–89 years who died of primary breast cancer in 2010 or 2011 were selected as cases (8288 cases). When possible, two controls were selected per case (15,202 controls) and were matched by date of birth and screening area.

**Results:**

Conditional logistic regressions showed a 38% reduction in breast cancer mortality after correcting for self-selection bias (OR 0.62, 95% CI 0.56–0.69) for women being screened at least once. Secondary analyses by age group, and time between last screen and breast cancer diagnosis were also performed.

**Conclusions:**

According to this England-wide case-control study, mammography screening still plays an important role in lowering the risk of dying from breast cancer. Women aged 65 or over see a stronger and longer lasting benefit of screening compared to younger women.

## Background

Following an evaluation of several randomised controlled trials (RCT)^1^ that showed an overall reduction in mortality from breast cancer in women undergoing mammography screening, the National Health Service Breast Screening Programme (NHS BSP) was launched in the United Kingdom (UK) in 1988. At the time, it aimed to offer free routine screening to every woman aged 50–64 once every three years. It now invites women aged 50–70, with an age extension to younger and older women (47–73 years) being trialled.^2^

Over the last thirty years, major advances have been made in the fields of cancer screening, treatment, and management (including effective adjuvant systemic therapies^3^ and two-view mammography^3,4^), with resulting lengthening of survival times after a breast cancer diagnosis.^5^ Despite recent reductions in breast cancer mortality, breast cancer is still the cancer with the highest incidence^6^ and the second most common cause of cancer death^7^ in females in the UK.

Case-control studies are a useful tool to evaluate screening programmes in settings where lack of equipoise would mean that RCTs would be unethical, or as in this case, where the RCTs have already been done, but there remains a need to ensure that the service is delivering the expected clinical benefit. Case-control studies also overcome some limitations associated with other observational designs by taking into account changes in cancer incidence and use of treatments over time and adjusting for any imbalances in other factors that could affect breast cancer mortality.

Taking as an example a case-control study^8^ that resulted in policy change within the NHS cervical screening programme by altering age at first screen and the screening interval, we designed a similar study focussing on the NHS BSP with the aim of:

- Evaluating the effect of mammography screening in the NHSBSP on breast cancer mortality
- Evaluating the effect of mammography screening on breast cancer incidence, and incidence of late stage disease
- Estimating overdiagnosis
- Analysing the interplay of early detection, pathology, and treatment on fatality of breast cancer.

The study protocol and results from two pilot studies have been published previously.^9–11^ This paper reports on the first objective above (breast cancer mortality), making use of England-wide data. Effects on incidence etc. will be reported in future papers.

## Methods

### Definition of cases and controls

As the main objective was to evaluate the effect of mammography screening on breast cancer mortality, cases were defined as women whose primary cause of death was breast cancer, who were diagnosed at age 47 years or older and died at age 89 years or younger in 2010–2011. We chose the lower limit of 47 as there is a major trial of screening in ages 47–49 ongoing,^2^ so substantial numbers of women have been screened in this age group. We chose the upper limit of 89 because above this age we would not expect a major effect of screening taking place mainly at ages 50–70, because we were less confident of the cause of death in the very old, and because screening is essentially aimed at preventing premature mortality, which one might reasonably interpret as death below age 90 years. Only diagnoses occurring after 1990 were included in the analysis. Their matched controls were women sampled from the general population of those invited for screening (99.9% of women eligible for screening in England^12^) and alive at the time of their corresponding case’s death. Controls may have been diagnosed with breast cancer, but not before their case’s date of diagnosis. Where possible, two controls were selected per case and matched on date of birth (within one month of the case’s) and screening area at date of diagnosis.

For the purposes of the statistical analysis, controls were assigned a date of pseudodiagnosis, equal to the diagnosis date of their corresponding matched case. To be eligible as a case or a control, a woman had to have had at least one invitation to screening prior to the date of diagnosis/pseudodiagnosis.

### Endpoints

The primary endpoint was to estimate, among those invited to breast screening, the effect of ever attending breast screening on mortality from breast cancer. Changes in this effect over time were also investigated. Secondary endpoints included the effect of measures of screening intensity, such as time between last screen and diagnosis/pseudodiagnosis, and their estimations in different age subgroups.

### Data selection and linkage

Cases were identified from the National Cancer Registration and Analysis Service (NCRAS) database accessed through the Office for Data Release of Public Health England (PHE). This database contains Office for National Statistics date and cause of death data. NHS Digital used the National Health Application and Infrastructure Services (NHAIS) system to identify matched controls and provided breast and cervical screening histories within.

We excluded any breast screens occurring outside the usual call/recall system of the national screening programme. All the screening histories of the study subjects were considered up to and including their date of diagnosis/pseudodiagnosis.

The data were processed according to the NHS Information Governance guidelines.^13^

### Sample size

Sample size calculations for the pilot study showed that, assuming an OR for breast cancer mortality of 0.7 and a number of discordant pairs of 33%, two controls per case with 800 breast cancer deaths and 1600 controls would confer more than 90% power to detect such an effect size at the 5% significance level using a two-sided test.^10^ As the data for this main phase encompassed the whole of England, we had ample power, not only for the primary outcome (8288 cases and 15,202 controls after exclusions), but also for subgroup analyses.

### Statistical analysis

Data were analysed using Stata version 13^14^ by matched (conditional) logistic regression with death from primary breast cancer as the outcome. Date of birth and screening area were accounted for by the matching process.

**Fig. 1:**
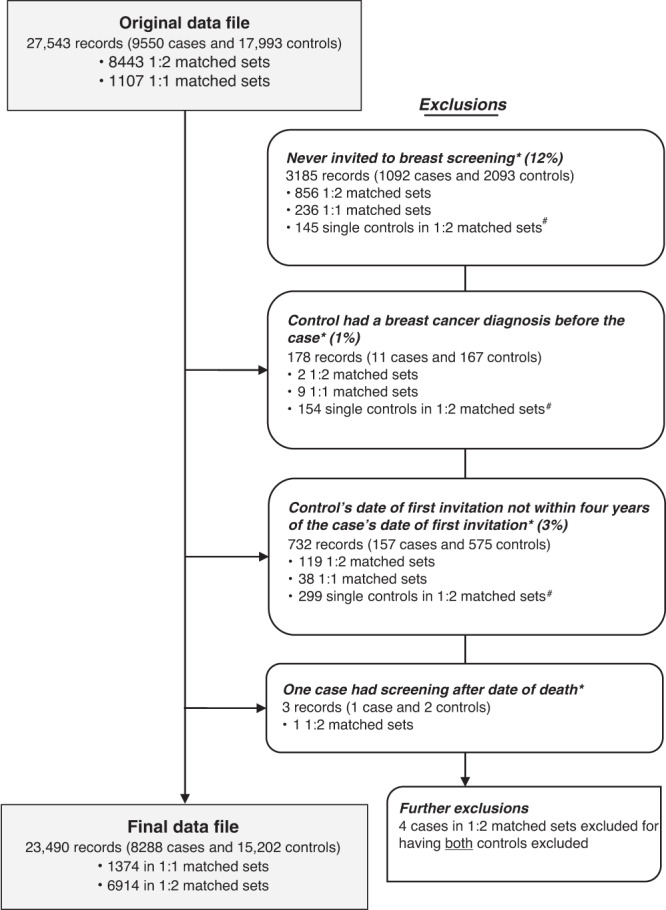
Study flow diagram. Asterisk indicates that these records were excluded for being in a 1:1 matched set where the case or the control was excluded or for being in a 1:2 matched set where the case or both controls were excluded. Hash indicates that these become 1:1 matched sets in the final dataset. Note: some records may be excluded for more than one reason.

Ineligible subjects were excluded (see Fig. 1). For some of these, this resulted in a matched set containing only a case, or only controls, which could then no longer be used in the matched logistic regression. Sensitivity analyses using unmatched logistic regression and controlling for age at diagnosis/pseudodiagnosis and screening area were performed on the same dataset with fewer exclusions; in this case, the inclusion criteria considered were the same, but the fact that a case or a control was excluded did not imply discarding that matched set.

Case-control studies used to evaluate population screening programmes are subject to a type of bias known as non-compliance or self-selection bias, which is based on the assumption that people who are already ill may be less likely to attend screening and those who do attend may be more health conscious, and therefore healthier, than those who do not take up the invitation. This may confer an artificially greater protective effect for screening, which was corrected in our analyses using a variant of the method by Duffy et al.^15^

The effect of self-selection bias was estimated using data available on cervical screening attendance for the women in the study, on the basis that any observed protective effect of cervical screening on breast cancer death cannot be due to cervical screening (which does not include breast examination) and is therefore likely to be caused by self-selection bias. In particular, the odds ratio (OR) uncorrected for self-selection is an estimate of the relative risk:

$$\gamma = \frac{{P({\mathrm{die}}\,{\mathrm{from}}\,{\mathrm{breast}}\,{\mathrm{cancer}}\;|\;{\mathrm{choose}}\;{\mathrm{to}}\;{\mathrm{attend}}\;{\mathrm{breast}}\;{\mathrm{screening}})}}{{P({\mathrm{die}}\;{\mathrm{from}}\;{\mathrm{breast}}\;{\mathrm{cancer}}\;|\;{\mathrm{choose}}\;{\mathrm{not}}\;{\mathrm{to}}\;{\mathrm{attend}}\;{\mathrm{breast}}\;{\mathrm{screening}})}}.$$

An unbiased estimate of the effect of screening on risk of dying from breast cancer would be (refer to Duffy et al.^15^):

$$\theta = \frac{{P({\mathrm{die}}\;{\mathrm{from}}\;{\mathrm{breast}}\;{\mathrm{cancer}}\;|\;{\mathrm{choose}}\;{\mathrm{to}}\;{\mathrm{attend}}\;{\mathrm{breast}}\;{\mathrm{screening}})}}{{P({\mathrm{die}}\;{\mathrm{from}}\;{\mathrm{breast}}\;{\mathrm{cancer}}\;|\;{\mathrm{not}}\;{\mathrm{invited}}\;{\mathrm{to}}\;{\mathrm{breast}}\;{\mathrm{screening}}\;{\mathrm{but}}\;{\mathrm{would}}\;{\mathrm{have}}\;{\mathrm{attended}}\;{\mathrm{if}}\;{\mathrm{invited}})}}.$$

The OR for death from breast cancer associated with attendance at cervical screening, i.e. the self-selection correction factor, can be considered an approximate estimate of the relative risk:

$$\varphi = \frac{{P({\mathrm{die}}\;{\mathrm{from}}\;{\mathrm{breast}}\;{\mathrm{cancer}}\;|\;{\mathrm{not}}\;{\mathrm{invited}}\;{\mathrm{to}}\;{\mathrm{breast}}\;{\mathrm{screening}}\;{\mathrm{but}}\;{\mathrm{would}}\;{\mathrm{have}}\;{\mathrm{attended}}\;{\mathrm{if}}\;{\mathrm{invited}})}}{{P({\mathrm{die}}\;{\mathrm{from}}\;{\mathrm{breast}}\;{\mathrm{cancer}}\;|\;{\mathrm{choose}}\;{\mathrm{not}}\;{\mathrm{to}}\;{\mathrm{attend}}\;{\mathrm{breast}}\;{\mathrm{screening}})}}.$$

Therefore, we obtain an estimate of *θ* by dividing *γ* by *φ*. The fundamental assumption here is that the populations choosing to attend or not to attend cervical cancer screening have the same risk of dying of breast cancer a priori as those choosing or not choosing to attend breast cancer screening. We do not assume that the effects of self-selection are the same in the two programmes. This is referred to as our first method of correction in the Results section.

As there is considerable uncertainty in the extent of self-selection, and of course decisions to attend at two separate screening programmes are likely to be confounded with each other, we also corrected for this using the method of Duffy et al.^15^. This method estimates the effect of participation in screening in those who would participate if invited as:

$${\mathrm{RR}}_2 = \frac{{p\gamma D_r}}{{(1 - \left( {1 - p} \right)D_r)}}$$

where *p* is the proportion of the invited population who participate in screening and D_r_ is the a priori relative risk of dying of breast cancer for someone who chooses not to attend compared to an uninvited general population member. We estimated D_r_ as 1.19 (95% CI 1.11–1.27), from the cohort study of Johns et al.^16^ Thus, this correction was based on a prospective estimate of the extent of self-selection bias in a cohort of 988,090 women in the NHS Breast Screening Programme. We estimated *p* as 73.4% from the annual report of the National Programme.^12^ This method, referred to as our second method of correction in the Results section, also yields an estimate of the effect of invitation to screening as follows:^15^

$${\mathrm{RR}}_1 = D_r\left( {p\gamma + 1 - p} \right).$$

More details on the methods are available in the published study protocol^9^ and pilot study analysis.^10^

## Results

The study dataset had a total of 9550 cases and 17,993 controls. There were 1107 sets with matching ratio 1:1 (1 case to 1 control) and 8443 sets with matching ratio 1:2 (1 case to 2 controls). Records of 1262 cases and 2791 controls (15% of the total) were excluded for various reasons before the statistical analysis (see study flow diagram in Fig. 1). This left a final dataset of 8288 cases and 15,202 controls, divided into 1,374 matched sets of size 1:1 and 6914 of size 1:2.

Sensitivity analyses using unconditional logistic regression were performed including subjects without a matched case or control, leaving us with 8479 cases and 16,794 controls.

Table 1 shows patient demographics and screening histories. Median age at first diagnosis was 64 years for both cases and controls and median age at death for cases was 71 years. Whilst the distributions of the number of screening invitations in the two study groups were comparable, differences can be noted in screening attendance, with 72% of the cases versus 82% of the controls attending their first screening invitation; 64% of the cases versus 76% of the controls attending their last screening invitation before diagnosis/pseudodiagnosis; and 21% of the cases versus 12% of the controls never being screened. Median time between last screen and date of diagnosis/pseudodiagnosis for compliers was also slightly longer for cases. From the data available on cervical screening history up to the date of diagnosis/pseudodiagnosis, it can be noted that 22% of the cases compared with 19% of the controls never had a cervical screen.

**Table 1:** Patient demographics and screening history by case-control status.

	Controls (*n* = 15,202)	Cases (*n* = 8288)
Patient demographics		
Breast cancer diagnosis and death		
Year of first diagnosis/pseudodiagnosis (Count, %)		
1990–1994	505 (3.3)	275 (3.3)
1995–1999	1481 (9.7)	792 (9.6)
2000–2004	2988 (19.7)	1630 (19.7)
2005–2011	10,228 (67.3)	5591 (67.5)
Age at first diagnosis/pseudodiagnosis (Count, %)		
47–54	2328 (15.3)	1241 (15.0)
55–59	2772 (18.2)	1493 (18.0)
60–64	2783 (18.3)	1530 (18.5)
65–69	2343 (15.4)	1303 (15.7)
70–74	2238 (14.7)	1222 (14.7)
75–89	2738 (18.0)	1499 (18.1)
Median age at first diagnosis/pseudodiagnosis in years (range)	64.4 (48.0–86.5)	64.6 (48.0–86.5)
Median age at death in years (range)	–	70.8 (49.6–88.4)
Patient screening history		
Breast screening history		
Number of screening invitations (Count, %)		
1	3700 (24.3)	2012 (24.3)
2	3486 (22.9)	1863 (22.5)
3	2859 (18.8)	1585 (19.1)
4	2257 (14.8)	1238 (14.9)
5+	2900 (19.1)	1590 (19.2)
Median age at first screening invitation in years (range)	523 (47.1–70.0)	52.5 (47.0–69.8)
Attendance at first screening invitation		
Did not attend	2772 (18.2)	2345 (28.3)
Attended	12,430 (81.8)	5943 (71.7)
Median age at last screening invitation in years (range)	61.8 (47.2–73.1)	61.9 (47.0–72.7)
Attendance at last screening invitation		
Did not attend	3583 (23.6)	2980 (36.0)
Attended	11,619 (76.4)	5308 (64.0)
Number of screens (Count, %)		
0 (Never screened)	1803 (11.9)	1741 (21.0)
1	3923 (25.8)	2117 (25.5)
2+	9476 (62.3)	4430 (53.5)
Median time between last screen and diagnosis/pseudodiagnosis (range)—among compliers	2.4 years (1 day–22.6 years)	2.7 years (0–22.2 years)
Median age at first screen in years (range)—among compliers	53.1 (47.1–72.1)	53.6 (47.2–70.6)
Median age at last screen in years (range)—among compliers	61.7 (47.3–73.2)	61.5 (47.3–72.8)
Cervical screening history		
Attendance at cervical screening (Count, %)		
Never screened	2818 (18.5)	1850 (22.3)
Screened	12,384 (81.5)	6438 (77.7)

Table 2 summarises the main results without and with correction for self-selection bias. Using data from cervical screening attendance, the self-selection correction factor was estimated to be 0.78 (95% CI 0.73–0.84). The primary endpoint, the association between attending one or more screens and death from breast cancer, had a resulting OR = 0.49 (95% CI 0.45–0.53) and, when corrected for self-selection, had OR = 0.62 (95% CI 0.56–0.69) by our first method and OR = 0.63 (95% CI 0.55–0.71) by our second. Using the second method, the estimate of the effect of invitation to screening was a 26% reduction in breast cancer mortality (OR = 0.74, 95% CI 0.68-0.81). The unmatched logistic regression on the larger dataset for sensitivity analyses showed a similar effect of screening on breast cancer mortality both before and after controlling for age at diagnosis/pseudodiagnosis and screening area (in both cases, uncorrected OR = 0.55, 95% CI 0.51–0.59).

**Table 2:** Results of the matched logistic regression evaluating the association between screening attendance and breast cancer mortality.

Exposure	Category of exposure	Controls (*n* = 15,202)	Cases (*n* = 8288)	OR (95% CI)	OR (95% CI) corrected for self-selection^a^	OR (95% CI) corrected for self-selection^b^
Ever screened	No	1803	1 741	1.00	–	–
	Yes	13,399	6 547	0.49 (0.45–0.53)	0.62 (0.56–0.69)	0.63 (0.55–0.71)

^a^Self-selection correction performed using our first method (variant of Duffy et al.^15^), with the OR of 0.78 associated with participation in cervical screening.

^b^Self-selection correction performed using our second method (Duffy et al.^15^).

In order to analyse changes of the effect of screening over time, we excluded women diagnosed before year 2000 (13% of the total records), which led to a corrected OR of 0.56 (95% CI 0.51–0.63) for the effect of ever attending mammographic screening on breast cancer mortality. Women diagnosed from year 2003 onwards had an even larger benefit from being screened (OR corrected by first method = 0.53, 95% CI 0.47–0.59). The estimated effect continued to increase as we restricted the year of diagnosis/pseudodiagnosis further in time (Supplementary Fig. 1).

Table 3 shows how the effect of screening varies depending on how much time has passed between a woman’s last screen and her diagnosis/pseudodiagnosis. Screen-detected cancers (assumed to be cancers diagnosed within three months of screening) showed a positive association with breast cancer fatality, after self-selection bias correction by our first method (OR = 1.93, 95% CI 1.68–2.22), while women screened in any other time interval were at reduced risk of dying from breast cancer. This was lowest for women screened in the last year (OR corrected by our first method = 0.19, 95% CI 0.17–0.23) and gradually increased, while still conferring a beneficial effect to screening, for women screened further back in time with respect to their date of diagnosis/pseudodiagnosis. Results using our alternative correction for self-selection were very similar (Table 3). Note that the time is from screening to diagnosis, not to death. The Table shows risk of subsequently dying of breast cancer increasing by the time between the screen and diagnosis/pseudodiagnosis.

**Table 3:** Results of the matched logistic regressions evaluating the association between time since last screening attendance and breast cancer mortality.

Exposure	Category of exposure	Controls (*n* = 15,202)	Cases (*n* = 8288)	OR (95% CI)	OR (95% CI) corrected for self-selection^a^	OR (95% CI) corrected for self-selection^b^
Time between last screen and diagnosis/pseudodiagnosis	Never screened	1803	1741	1.00	–	–
	0 ≤ 3 months	925	1383	1.50 (1.33–1.70)	1.93 (1.68–2.22)	1.91 (1.64–2.24)
	3 ≤ 12 months	2172	407	0.15 (0.13–0.18)	0.19 (0.17–0.23)	0.19 (0.15–0.24)
	12 ≤ 24 months	2573	788	0.24 (0.22–0.27)	0.31 (0.27–0.35)	0.31 (0.26–0.36)
	24 ≤ 36 months	2345	992	0.35 (0.32–0.40)	0.45 (0.39–0.51)	0.45 (0.38–0.52)
	36 ≤ 48 months	639	344	0.49 (0.42–0.57)	0.61 (0.51–0.73)	0.63 (0.52–0.75)
	48 ≤ 60 months	429	245	0.54 (0.45–0.66)	0.70 (0.57–0.86)	0.69 (0.55–0.86)
	>60 months	4316	2388	0.67 (0.60–0.74)	0.85 (0.75–0.96)	0.86 (0.74–0.99)

^a^Self-selection correction performed using our first method (variant of Duffy et al.^15^), with the OR of 0.78 associated with participation in cervical screening.

^b^Self-selection correction performed using our second method (Duffy et al.^15^).

**Fig. 2:**
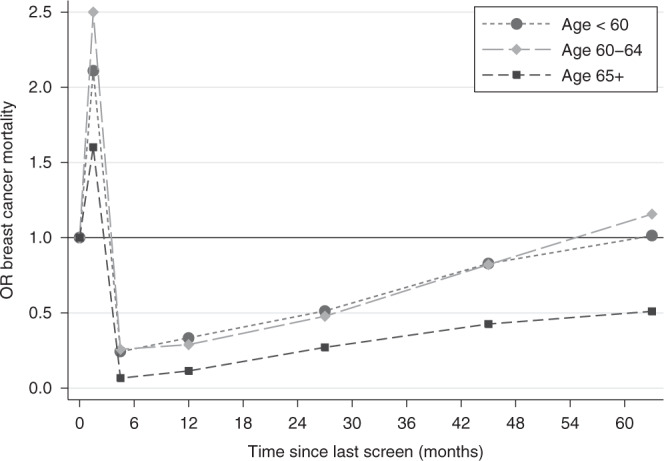
Graph of the corrected odds ratios (using our first method of correction) for breast cancer mortality versus time between last screen and diagnosis/pseudodiagnosis by age category. Note: the coordinates on the *x*-axis are the midpoints of the time intervals: 0–3, 3–6, 6–18, 18–36, 36–54 and 54–72 months.

A similar analysis is shown in Table 4 and Fig. 2 for different time intervals after stratifying for three different age categories at diagnosis/pseudodiagnosis (younger than 60 years, between 60 and 64 years, and 65 years or older). The results show that the protective effect of a screen is greater and lasts longer in the oldest group. The benefit of attending screening in the three years prior to diagnosis/pseudodiagnosis, the recommended interval for screening in the NHS BSP, is shown in the final row of Table 4, and shows close to a halving of risk with screening within the recommended interval, following self-selection correction by our first method (OR = 0.51, 95% CI 0.46–0.57). Results using our second method of correction were very similar to those using the first (Supplementary Table 1). The estimated effect of invitation to screening within the last 36 months using our second method was a 33% reduction in breast cancer mortality (OR = 0.67, 95% CI 0.61–0.73).

**Table 4:** Results of the matched logistic regressions evaluating the association between time since last screening attendance and breast cancer mortality, stratified by age at diagnosis/pseudodiagnosis.

Exposure	Category of exposure	Age 47–59 (*n* = 7834)		Age 60–64 (*n* = 4313)		Age 65–89 (*n* = 11,343)		All ages (*n* = 23 490)	
		OR (95% CI)	OR (95% CI) corrected for self-selection^a^	OR (95% CI)	OR (95% CI) corrected for self-selection^a^	OR (95% CI)	OR (95% CI) corrected for self-selection^a^	OR (95% CI)	OR (95% CI) corrected for self-selection^a^
Time between last screen and diagnosis/pseudodiagnosis	Never screened	1.00	–	1.00	–	1.00	–	1.00	–
	0 ≤ 3 months	1.64 (1.39–1.94)	2.11 (1.76–2.52)	1.98 (1.52–2.57)	2.50 (1.91–3.28)	1.22 (0.93–1.60)	1.60 (1.21–2.13)	1.50 (1.33–1.69)	1.93 (1.67–2.22)
	3 ≤ 6 months	0.19 (0.14–0.25)	0.24 (0.18–0.32)	0.18 (0.12–0.27)	0.26 (0.17–0.39)	0.05 (0.03–0.10)	0.07 (0.04–0.12)	0.14 (0.11–0.17)	0.18 (0.15–0.23)
	6 ≤ 18 months	0.27 (0.23–0.32)	0.33 (0.28–0.40)	0.22 (0.17–0.29)	0.29 (0.22–0.38)	0.09 (0.07–0.12)	0.11 (0.08–0.15)	0.19 (0.17–0.22)	0.24 (0.21–0.28)
	18 ≤ 36 months	0.41 (0.35–0.47)	0.51 (0.44–0.61)	0.38 (0.30–0.48)	0.48 (0.38–0.61)	0.22 (0.18–0.26)	0.27 (0.22–0.34)	0.32 (0.29–0.36)	0.40 (0.36–0.46)
	36 ≤ 54 months	0.66 (0.51–0.85)	0.83 (0.64–1.07)	0.72 (0.51–1.01)	0.82 (0.58–1.16)	0.34 (0.27–0.42)	0.43 (0.34–0.54)	0.50 (0.43–0.58)	0.62 (0.53–0.73)
	54 ≤ 72 months	0.80 (0.56–1.13)	1.01 (0.71–1.45)	0.95 (0.65–1.40)	1.16 (0.78–1.71)	0.35 (0.28–0.44)	0.51 (0.40–0.65)	0.54 (0.46–0.64)	0.74 (0.62–0.88)
	>72 months	0.87 (0.57–1.35)	1.07 (0.70–1.64)	1.02 (0.72–1.44)	1.34 (0.94–1.90)	0.57 (0.50–0.64)	0.71 (0.62–0.82)	0.69 (0.62–0.76)	0.86 (0.76–0.98)
	0–36 months	0.50 (0.44–0.56)	0.63 (0.55–0.73)	0.47 (0.39–0.57)	0.60 (0.49–0.74)	0.27 (0.23–0.32)	0.34 (0.29–0.41)	0.40 (0.37–0.44)	0.51 (0.46–0.57)

^a^Self-selection correction performed using our first method (variant of Duffy et al^15^), with the OR of 0.78 associated with participation in cervical screening.

## Discussion

Despite the many improvements in treatments, diagnostic procedures and technologies over the last thirty years, and changes in baseline rate of breast cancer mortality, our data showed an overall reduction in the risk of dying from breast cancer of ~38% for women attending at least one mammography screen, after adjusting for self-selection bias. This is in line with the results obtained from the pilot phase of the study,^10^ in which a mortality reduction of 39% was seen for women attending screening in London (deaths occurring in 2008–2009). Using the same calculation method as in the review by the Independent UK Panel on Breast Cancer Screening UK Independent Review,^17^ this would correspond to approximately nine breast cancer deaths prevented for every 1,000 women attending screening at ages 50–69 years, larger than but in the same general scale as the six deaths estimated from the UK Independent review.

It should be noted that there is a wide range of estimates of the absolute mortality benefit of mammography screening^18–21^ some finding considerably smaller benefits than above. The size of the estimated effect depends on sources used and assumptions made. However, it has been shown to depend more crucially on whether the effect pertains to screening per se or to invitation to screening only, and on the timescale envisaged.^22^ Screening prevents deaths not this year or next, but 5, 10, 15 or 20 years from now. Considering the effect of screening on 10-year mortality will considerably underestimate the absolute benefit. Nevertheless, it should be acknowledged that while the body of evidence, randomised and observational, points to a substantial reduction in breast cancer mortality with screening, there is sufficient variation that different views are still possible.

Our first method of correction for self-selection caused a decrease of about 25% in the estimated protective effect of screening for women having at least one mammogram. The second method yielded similar results. This is a greater correction than the one estimated in the pilot phase,^10^ where self-selection only played a minor role, despite the fact that the final risk reduction is very similar. London has a lower coverage than the rest of England for both breast and cervical screening, which is largely explained by factors like deprivation and ethnicity.^23^ Such variations in coverage might be one of the causes for the different impact of self-selection between the two phases of the study. For example, a larger population of non-participants, such as in London, may be less different in health status than a smaller population. In the Swedish two-County trial,^24^ where only 15% of the population were non-participants, the rate of death from breast cancer in this population was very high. It is also worth noting that, during the early 21st century, breast screening attendance was rapidly increasing in London, and the socioeconomic gradient in attendance was reducing with time nationally.^25,26^

Case-control studies tend to give higher estimates of benefit than other evaluations, largely because they assess the effect of actually being screened rather than simply being invited to screening.^19,27^ It should be noted that with our second correction for self-selection bias, we were able to estimate the effect of invitation, giving a 26% breast cancer mortality reduction, similar to the effect observed in the randomised trials in this age group and to the prospectively estimated effect of a 25% reduction in the Copenhagen screening programme.^28^ As a comparison, in the review by the Independent UK Panel on Breast Cancer Screening,^17^ a meta-analysis of 11 RCTs found that the relative risk reduction of breast cancer mortality for women invited to screening was 20%. Furthermore, in the same report, the panel stated that the case-control studies that they had analysed seemed to inflate the benefit of screening compared to the trials and postulated that this may have been caused by some residual bias unaccounted for by the authors. We believe that our adjustments for self-selection bias has largely accounted for this and that the greater effect of screening in this study is due to technical improvements in mammography since the RCTs were carried out, accompanied by improved treatment and strong quality assurance measures in the NHS BSP.^11^

The greater benefit of screening observed for women diagnosed after year 2000 was similar to the pilot study,^10^ but here we were able to restrict the analysis to later years of diagnosis and see the benefit getting larger (data not shown). We could conjecture that this improvement was due to the introduction of better procedures in the NHS BSP, such as two-view mammography at every attendance in year 2000^4^; however, there may be a bias in comparing different times since diagnosis as we only have data on deaths in years 2010–2011. In the first place, cases diagnosed before 2000 have a long survival by definition, and there might therefore be an over-representation of screen-detected cancers. In other words, it is more likely that a case diagnosed before year 2000, for example, who had a breast cancer for more than 10–11 years before dying from it, had a screen-detected cancer rather than a symptomatic one. This confers a bias against screening in the analysis of cancers diagnosed prior to the year 2000. In the second place, there will be a bias in favour of screening if the analysis is restricted to cancers diagnosed within a short time before death, i.e. if we only consider women (pseudo)diagnosed a few years before 2010–2011. We are therefore unable to make any definitive conclusions on the impact of any improvements in the NHS BSP over time.

As shown in RCTs of breast screening,^24^ measures of the benefit of screening are largely influenced by the consequent reduction in mortality from symptomatic cancers. This is due to the fact that screen-detected cancers (defined as the ones diagnosed within three months of a screen), despite being less fatal overall, represent a larger proportion of the cancer-related deaths in the immediate period after a screen as it can be seen from the spike in excess mortality in Fig. 2.

The duration of the benefit of attending screening appears to be greater in older women (Table 4 and Fig. 2). Women aged 65 or more see the greatest and longer lasting benefit, which might suggest that they could be screened less often than younger women. This result is in agreement with the impact of ageing on breast cancer biology^29^ and is also potentially important in light of the recent incident in the NHS BSP, where a number of women aged 69 and 70 years did not receive the scheduled invitation to their last screening appointment.^30^ The exact number affected has been debated but an Independent Review concluded that 5000 women were not invited as scheduled, and that a further 62,000 could be interpreted as having missed their final invitation as defined in the service specification.^30^ Our findings suggest that the effect of a delayed screen in older women has a lesser consequence for increased risk of breast cancer mortality than it would have had in younger women. While three years is a longer interval than other programmes in Europe and North America, and further slippage of the interval should be avoided if at all possible, these results could also be used as guidelines for screening units at times of capacity constraints, with the provision that all women receive an opportunity for a final screen around or shortly after age 70. There is interest in stratified screening and these results may inform further thinking on this subject.

A limitation of the study is the retrospective design and the potential for self-selection bias. We have corrected for this in two different ways and for one of these, an effect of invitation to screening was derived which was consistent with trials results and prospective studies for this age group. However, it must be acknowledged that there remains some uncertainty about the extent of self-selection bias. Furthermore, case-control studies for cancer screening programmes are subject to an inherent type of anti-screening bias known as screening opportunity bias.^27^ As most of the controls do not have a breast cancer diagnosis, the only way they can be exposed to screening is if they attended a mammography appointment in the past. Cases, on the other hand, may have had a screen in the past, but some of them will also have an additional screen for when their cancer was diagnosed. This induces an artificially higher retrospective probability of screening exposure among cases. Screening opportunity bias was corrected for in the pilot study,^10^ where a 10–15% increase in mortality reduction was seen following this, but here we preferred to keep a conservative approach and not adjust for it. To minimise biases with respect to age and opportunity to be screened, we matched very closely for age. This meant that in 1107 cases out of 9550, we could only find one control.

Although the effect of the NHS BSP in preventing breast cancer mortality has been assessed several times,^31–34^ we are aware of only one other case-control study conducted using national data.^34^ The latter relies on data up to year 2005 (diagnoses and deaths took place between 1991 and 2005), while ours uses more recent data up to year 2012, arguably more in the epoch of effective adjuvant systemic therapies. It is of interest that our more recent case base shows similar results in terms of the reduction in risk of breast cancer death with screening. In any case, we suggest that it would be of interest to repeat this type of analysis for years thereafter, to ensure that the programme continues to deliver its aims even with the introduction of new diagnostic technologies (e.g. digital mammography). Before the establishment of the NHS BSP in 1987, it was suggested that a routine case-control assessment could and should be part of an ongoing evaluation of a mass screening programme.^35^ For this reason, we believe that this exercise should be held on a two-yearly basis.

The results of further national case-control studies (1) evaluating the effect of the NHS BSP on breast cancer incidence and incidence of late stage disease, (2) estimating overdiagnosis, and (3) analysing the interplay of early detection, pathology and treatment on fatality of breast cancer will be published shortly.

To conclude, this study showed that the breast screening programme in England continues to play an important role in the control of breast cancer. The effect of screening within the NHS BSP in England is stronger and longer lasting in women aged 65 or over, but it remains highly relevant for younger women.

## Supplementary information

Supplementary Materials

## Data Availability

Data were saved on the servers of the Barts Cancer Institute, Queen Mary University of London, in a folder with restricted access to D.P. A clean, anonymised version of the data was produced and made available to R.M., A.D. and S.W.D. with restricted access to the staff of the Policy Research Unit in Cancer Awareness, Screening and Early Diagnosis at Queen Mary University of London. The data were obtained via the Office for Data Release at Public Health England. We do not have authority to share the data with others, but requests for access to data will be forwarded to the Office for Data Release.
