# Supplementary material for: A case-control study to evaluate the impact of the breast screening programme on mortality in England
Source: Br J Cancer. 2020 Nov 23;124(4):736–43. doi: 10.1038/s41416-020-01163-2 (PMC7884709; doi:10.1038/s41416-020-01163-2)
Supplement: Supplementary file 1 — Supplementary Materials [file 41416_2020_1163_MOESM1_ESM.docx]

**A case-control study to evaluate the impact of the breast screening programme on mortality in England**

Running title: *The impact of breast screening on mortality*

Roberta Maroni (MSc)^1^*, Nathalie J Massat (PhD)^1^*, Dharmishta Parmar (BA (Hons))^1^, Amanda Dibden (MSc)^1^, Jack Cuzick (PhD)^1^, Peter D Sasieni (PhD)^2^**, Stephen W Duffy (MSc)^1^†**

**Supplementary Figure 1.** Graph of the corrected odds ratios (using our first method of correction) for breast cancer mortality versus year of diagnosis/pseudodiagnosis.

Note: the coordinates on the *x*-axis represent intervals of time until the year 2011, e.g. 2000+ is ‘from 2000 to 2011’. The grey vertical bars represent the 95% confidence intervals.


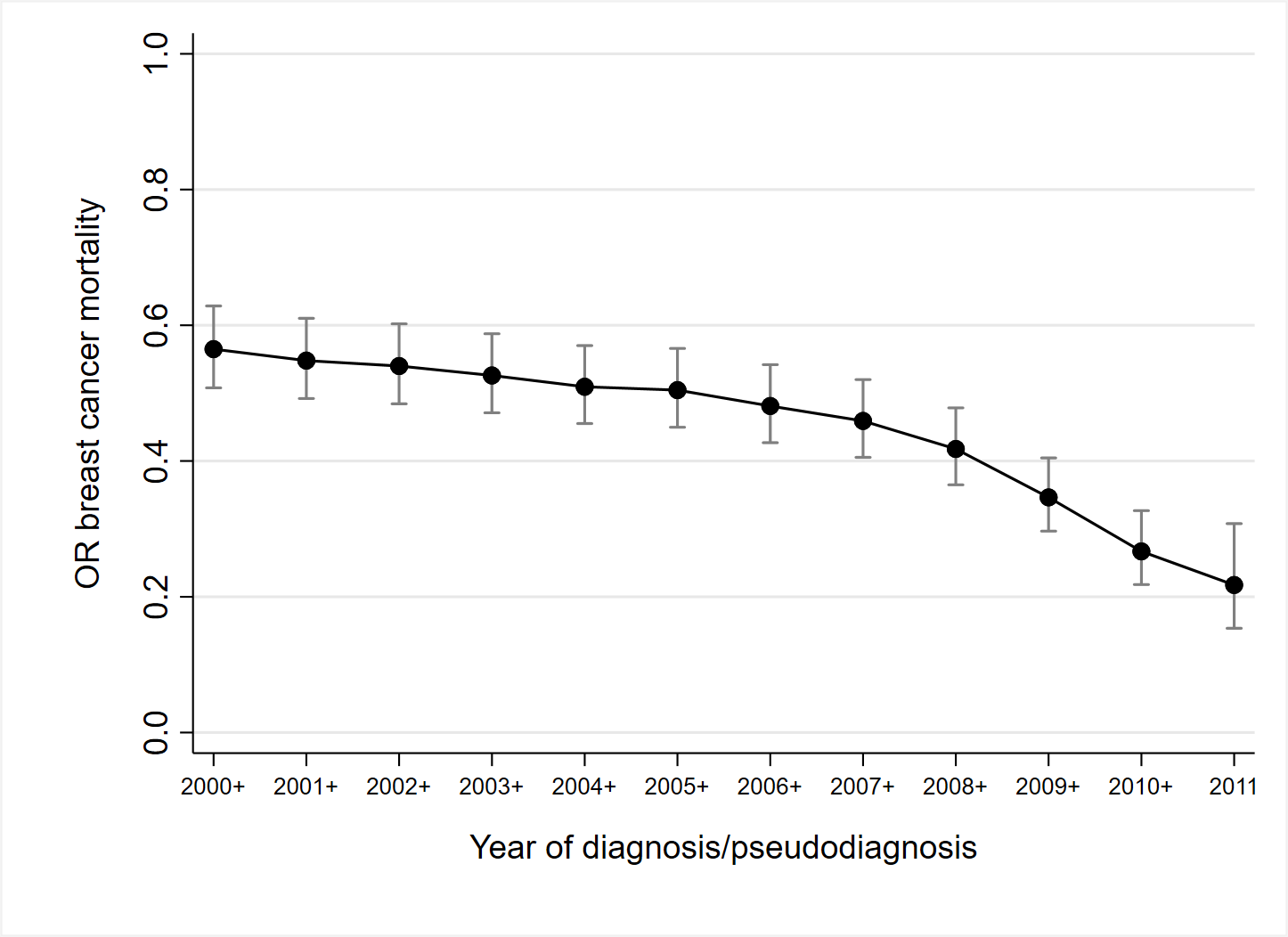


**Supplementary Table 1.** Results of the matched logistic regressions evaluating the association between time since last screening attendance and breast cancer mortality, stratified by age at diagnosis/pseudodiagnosis, corrected for self-selection bias using our second method of correction.

| **Exposure** | **Category of exposure** | **Corrected ORs and 95% CIs by age group** | | | |
| --- | --- | --- | --- | --- | --- |
|  |  | **Age 47-59**  **(n = 7 834)** | **Age 60-64**  **(n = 4 313)** | **Age 65-89**  **(n = 11 343)** | **All ages**  **(n = 23 490)** |
| Time between last screen and diagnosis/pseudodiagnosis | Never screened | - | - | - | - |
|  | 0≤3 months | 2.10  (1.72-2.54) | 2.53  (1.91-3.35) | 1.56  (1.16-2.08) | 1.92  (1.64-2.24) |
|  | 3≤6 months | 0.24  (0.17-0.33) | 0.23  (0.15-0.35) | 0.06  (0.03-0.12) | 0.18  (0.14-0.23) |
|  | 6≤18 months | 0.35  (0.28-0.42) | 0.28  (0.21-0.38) | 0.12  (0.08-0.16) | 0.24  (0.20-0.29) |
|  | 18≤36 months | 0.52  (0.43-0.63) | 0.49  (0.37-0.63) | 0.28  (0.22-0.35) | 0.41  (0.35-0.48) |
|  | 36≤54 months | 0.84  (0.64-1.11) | 0.92  (0.64-1.32) | 0.43  (0.34-0.56) | 0.64  (0.53-0.77) |
|  | 54≤72 months | 1.02  (0.71-1.48) | 1.21  (0.81-1.81) | 0.45  (0.35-0.58) | 0.69  (0.57-0.84) |
|  | >72 months | 1.11  (0.71-1.73) | 1.30  (0.91-1.87) | 0.73  (0.62-0.86) | 0.88  (0.76-1.02) |
|  | 0-36 months | 0.64  (0.54-0.75) | 0.60  (0.48-0.75) | 0.35  (0.28-0.42) | 0.51  (0.44-0.59) |
